# Relationship of Salivary Cortisol Level With Severe Depression and Family History

**DOI:** 10.7759/cureus.11548

**Published:** 2020-11-18

**Authors:** Qudsia U Khan

**Affiliations:** 1 Physiology, Combined Military Hospital (CMH) Lahore Medical and Dental College, Lahore, PAK

**Keywords:** depression, stress, salivary cortisol, family history

## Abstract

Salivary cortisol level is considered a prominent depression biomarker, as saliva induces less stress as compared to blood. The study was performed in the physiology department, Sheikh Zayed Medical Institute Lahore from April 2015 to December 2015. Sixty participants, including 14 (46.67%) males and 16 (53.33%) females, in each non-depressive and depressive group of over 17 years of age, were undertaken. The depression diagnostics were performed both outdoors and in clinics and confirmed with the standards of the Diagnostic and Statistical Manual of Mental Disorders and Beck’s Inventory. Saliva specimens were collected and processed for enzyme-linked immunoassay (ELISA), and absorbance was calculated on a microtiter plate reader. The statistics with the Statistical Package for the Social Packages (SPSS) 26.0 (IBM Corp., Armonk, NY) show that patients of the non-depressive category exhibited mean ages 35.73±6.89 years and 39.10±6.89 years in the depressive group (p-value: 0.178). The mean cortisol level was 1.46±0.91 ng/ml among non-depressive and 2.23±1.69 in depressive patients (p-value: 0.031). The mean depression score among non-depressive was 5.73±4.05 and 52.03±5.08 while there was no statistical difference in the mean height and weight of subjects in normal and depressive patients (p-value: 0.0001). Meanwhile, the mean cortisol level was 1.46±0.91 ng/ml among non-depressive patients, while it was 2.23±1.69 in the depressive group, with no statistical difference in mean ages (p-value: 0.031). These findings proved the cortisol level directly linked with severe depression and useful for depression diagnostics and management.

## Introduction

Depression is one of the most common mental disorders across the globe and affects all races, nations, cultures, and geographic locations. It is characterized by grief, the sensation of loneliness, tiredness, ill-temper, and guilt, fewer interests in routine activities, and distressed appetite, sleep, and concentration [1]. About 350 to 355 million people are affected throughout the world by depression comprising both males and females of all ages. About 16% of the population might have experienced depression once in their lives [2]. Internationally, the World Health Organization (WHO) has stated that stress is the prime source of major health issues [3]. In Pakistan, depression is affecting 44%, with about 50 million people of the overall population affected. Its occurrence is much greater in females at 57.5% and 25% in males [4].

The pathophysiology of depression associates with an imbalance in chemical neurotransmitters, including cortisol, serotonin, and noradrenaline, accompanied by structural variations such as neuronal atrophy and brain denaturation [5]. Depression has been found to be linked directly with a higher level of cortisol, also called the stress hormone. It is the most significant glucocorticosteroid produced in the cortex of the adrenal glands. The depression response encompasses the activation of the hypothalamic-pituitary-adrenal (HPA) axis and the sympathetic nervous system [6]. Any psychological or physical risk to homeostasis activates the release of corticotrophin hormone in the hypothalamus and eventually increases steroid hormonal levels, including cortisol in the saliva and bloodstream [7].

In a very short time, cortisol supports in fulfilling the stress demands through activating stored energy and contributes to recovery from anxiety by inhibiting more discharge of corticotrophin-releasing hormone [8]. On the other hand, enduring stress endorses the maladaptive running of the HPA axis, which, sequentially, might damage immune function, modify cardiovascular regulation, and compromise metabolism [9]. Major depression is linked to higher cortisol levels, and influence negatively the whole human body. Such elevation seems to be determined by the stress-induced hypersecretion of corticotrophin-releasing hormone besides the failure of negative feedback regulation to restrict the cortisol response [10].

The cortisol level in saliva has become a striking subject in recent years [11]. The amount of cortisol is higher in saliva as compared to serum, thus, the salivary cortisol level provides a better understanding of related depression [12]. Cortisol is a glucocorticoid and quantifiable within the saliva, urine, and plasma using cortisol assays for observing the effects of chronic and acute depression. In chronic depression, variations in cortisol levels are more conspicuous while acute depression is characterized by catecholamine variations [13]. Hence, the salivary cortisol level is more valuable as compared to serum cortisol for the valuation of stress and depression [14].

The most consistent and influential depression predictors include major recent life events, personal depression episodic history, and positive history of depression within the family [15]. Especially, people with major life events in their recent past are 2.5 to 12 times more prospective to develop depression. Likewise, people having first-degree family members suffering from stress are 2.8 to 10 times more probable for developing severe stress. Moreover, about 40 to 60 percent of people enduring a first-lifetime depression episode may acquire another with escalating more risks [16]. Salivary cortisol along with the family history is the emerging biomarker to recognize the level of depression and stress. This comparative and cross-sectional study was designed to identify the cortisol role in controlling depression. It has also compared the salivary cortisol levels in depressed and non-depressive patients and observed the link between depression development and family histories.

## Materials and methods

Study design and population

This comparative and cross-sectional study of eight (8) months duration was conducted in the department of physiology, Sheikh Zayed Federal Postgraduate Medical Institute Lahore from April 2015 to December 2015 jointly with Punjab Institute of Mental Health Lahore, with the authorization of the administration of the respective department. Each non-depressive and depressive group comprised those aged more than 17 years. After taking consent and signing a consent form, the purpose of doing this research and how the sample will be taken were explained. The diagnostics of depression during outdoor examination by psychiatrists and clinically confirmed by indicative criteria through the Diagnostic and Statistical Manual of Mental Disorders, 4th Edition, and Beck’s Inventory and acutely depressed patients satisfying the diagnostic criteria of Beck’s scoring were included. Since comorbidities were excluded, all the patients with confirmed hyperaldosteronism, Cushing syndrome, and under treatment for exogenous steroid diseases were excluded.

Data collection

Convenient and non-probability sampling was performed by keeping a sample size of 30 in each group, with a 5% significance level and a 90% power of the study through the following formula.

The total samples were 60 divided into two groups of 30 patients consistent with Beck’s score of depression. The scoring of depression was done using standardized criteria by Beck’s Depression Inventory. A score between 1 and 10 was considered normal while a more than or equal to 40 score was marked as severe depression. The questions were standardized. A questionnaire was designed for data collection related to the overall physical fitness and family history of depression, diagnosed by clinicians. Personal data was recorded such as name, weight, height, age, employment, education, and marital status. The family and friends could also help the subjects in answering questions like sleeping and eating routines.

Specimen sampling

About 4-5 ml of saliva sample was collected in a clean glass tube, as required, to prepare a replica. Afterward, the subjects were asked to take 5 cc of normal salt into the mouth for a little while without forcefully rinsing before eating, drinking, or brushing teeth and to rinse it out for sample collection. The samples were stored at 4°C for about 24 hours. The centrifugation was performed on all saliva samples at 20,000 revolutions per minute (rpm) for 10 minutes and shifted to the secondary tube carefully, along with labeling them and stored at -20ºC for 30 days till further testing was performed.

Reagent preparation and assay performance

The freeze specimens were taken out for melting and centrifugation. The supernatants were collected and decanted in the newly labeled tubes. The working solutions were prepared of the cortisol-HRP (horseradish peroxidase) conjugate and washed the buffers. The required microwell strips were opened and 50 µl of each calibrator, control, and specimen sample was pipetted into correspondingly labeled wells in the form of replicas. One hundred µl of the conjugate working solution was pipetted into each well and kept in shaking incubation at 200 rpm at room temperature for 45 minutes. All wells were washed three times using 300 µl diluted washing buffer per well and covered the plate tightly with absorbent paper and ensured its dryness. One-hundred fifty µl of 3,3′,5,5′-tetramethylbenzidine (TMB) substrate was pipetted into every well in specific pauses and kept on a shaking incubator at room temperature for 15 to 20 minutes till it attains dark blue color with desired optical density (OD). The stopping solution was pipetted 50 µl into every well with similar intervals. The microwell plate’s reader was noted at 450 nm after 20 minutes of adding the stopping solution.

Salivary cortisol estimation

Salivary cortisol level was estimated by Cortisol ELISA Kit AccuDiag TM (Ref 7101-38). The enzyme immunoassay works on the principle of competitive binding. The vital reagents are obligatory for the reaction comprised of antibodies, native antigens, and enzyme-antigen conjugates. The competitive reaction proceeds between unlabeled antigen within standards and control patient samples and labeled enzyme antigen or conjugate on the same degree of binding sites on the antibody inside the microwell plate. The washing and pouring detached the unbounded constituents. After washing, the substrates of the enzymes were added. The enzyme reaction was completed by adding the stopping solution. The absorbance was calculated on a microtiter plate reader in which wash intensity was inversely proportional to cortisol proportion. A collection of standards was considered for plotting a typical curve to read and control the amount of cortisol directly.

Statistical analysis

The statistical analysis was performed on the Statistical Package for the Social Packages (SPSS) 26.0 (IBM Corp., Armonk, NY). The average ages, weights, heights, and related quantitative values were analyzed on the basis of means and standard deviations while qualitative values, including marital status, family history, and severity of depression, were measured on the basis of pie charts, frequency, and percentage. The verification of the mean difference in cortisol levels was performed related to a few significant risk factors like depression, age, height, and weight. The Kolmogorov Smirnov test was applied to check the normality of all variables and determine the proper statistics. With the help of the Mann-Whitney U test, the abnormal data were evaluated while the independent t-test was used for normally distributed data. The significant p-value was considered less than or equal to 0.05.

## Results

Concentration of salivary cortisol

In the non-depressive category, the patient’s mean ages were 35.73±6.89 years while in depressive patients, it was recorded as 39.10±6.89 with no statistical difference in mean ages (p-value= 0.178). Meanwhile, the mean cortisol level was 1.46±0.91 ng/ml among non-depressive patients while in the depressive group, it was recorded as 2.23±1.69. The mean level of cortisol was statistically significantly higher in depressive patients as compared to normal subjects (p-value= 0.031) as illustrated in Table 1.

**Table 1:** Comparison of the study participant’s ages and cortisol level

Age (Years)	Study Participants	Mean	S.D	Minimum	Maximum	P-Value
	Non-depressive	35.73	6.89	20.00	48.00	0.178
	Depressive	39.10	11.65	18.00	70.00	
Cortisol Level	Non-depressive	1.46	0.91	0.36	4.33	0.031
	Depressive	2.23	1.69	0.35	9.89	

The mean depression score of non-depressive patients was 5.73±4.05 while in depressive patients, it was 52.03±5.08. There was no statistical difference in the mean height and weight of subjects in normal and depressive patients (p-value=0.0001) (Table 2).

**Table 2:** Depression score of study participants IQR: interquartile range

Depression Score	Study Participants	Mean	S.D	Minimum	Maximum	Median (IQR)^a^	P-Value
	Non-depressive	5.73	4.05	1.00	16.00	5 (5.50)	< 0.0001
	Depressive	52.03	5.08	41.00	62.00	52(7.25)	

The mean cortisol level in the control men group was higher (1.81±1.02) as compared to non-depressive women (1.14±0.67), with a significant p-value < 0.05. Furthermore, the mean cortisol level in patients with a family history of depression was considerably greater (2.38±1.82) as compared to negative family history (1.58±1.06) with a significant p-value < 0.05. The mean level of cortisol was statically identical in both married and unmarried subjects (Table 3).

**Table 3:** Statistical outcomes of gender, family history, and marital status

Attributes	Study Participants	Non-depressive		Depressive		
		Mean ± SD	p-value	Mean ± SD	p-value	
Sex						
	Men	1.81±1.02	0.043	2.46±1.74	0.488	
	Women	1.14±0.67		2.02±1.67		
Family History	Yes	2.02±1.15	0.259	2.44±1.94	0.449	
	No	1.39±0.88		1.95±1.32		
Marital Status	Married	2.31±1.53	0.402	2.31±1.53	0.80	
	Unmarried	1.37±0.72		2.18±1.81		

## Discussion

Cortisol is the principal glucocorticoid that is produced and secreted by the adrenal cortex. It influences the metabolism of carbohydrates, fat, and protein; the integrity of myocardial and muscle maintenance; and the suppression of allergic and inflammatory activities [17]. Interaction of environmental and genetic factors along with the lifestyle of the patients can affect the level of stress and depression [18]. The current meta-analysis established that salivary cortisol level in depressed patients was significantly higher as compared to healthy controls.

Moreover, the samples administered by the ELISA technique exhibited higher salivary cortisol levels in stressed patients as compared to controls, with significant differences. Less knowledge of the other approaches might be because of the low proportion of reported studies. The anticipation of depression is an important stimulus for the adrenal cortex to release cortisol. The assessment of salivary cortisol concentration permits determining the swift variations in the adrenocortical events [19]. So, it has been recommended that the salivary cortisol level is directly associated with the depression level.

Greenwood and Shutt stated in their study on animals that the salivary cortisol assessment was verified as useful for measuring depression [20]. They found a significant increase in salivary cortisol as a reaction to the transportation of goats. In the current study, patients in the non-depressive category exhibited mean ages of 35.73±6.89 years with 39.10±6.89 years in the depressive with no statistical difference (p-value=0.178). The mean cortisol level assessed was 1.46±0.91 ng/ml among non-depressive patients and 2.23±1.69 in depressive, with no statistical difference (p-value=0.031).

An identical study performed by Ghasempour et al. uses a similar method for saliva sampling for cortisol in children [21], which increasingly grows during the growth of the cavity and extends to the highest ratio while placing the base materials. They found that the anxiety and pressure on the children reduced during the restoration placement and at the start of treatment. The relationship between depression and salivary cortisol was also found to be substantial by Botelho et al. [22]. In this study, it was found that the salivary cortisol levels and stress scores indicate self-supposition, as the responding children reported the nature of depression similar to our results.

## Conclusions

In conclusion, in this Pakistan-based population study, we found strong evidence of higher salivary cortisol levels in severe depression as compared to non-depressed subjects. This relationship is inclined by the family history that included prominent health factors. While selecting patients for the study, those patients who were taking antidepressants were excluded. These findings recommend that cortisol level may comprise a biological index of severe depression and add an element to the diagnostics and management of attitude and depression complaints. It also shows that the potential significance of a long-time family history of depression beyond several generations has a positive impact on depressed patients and their effective treatment. The specificity of the transfer of depression across the generations recommends that it might be homogeneous for further biological marker researches. Furthermore, the statistical data have the potential to support the recognition of multiple diseases surrounded by depression. Finally, such findings might also deliver significant information on the mechanisms of depression disorders, predominantly the role of the cortisol section in stress reactivity that might stimulate these conditions.
